# New York County Veterinary Medical Association

**Published:** 1896-01

**Authors:** J. E. Ryder

**Affiliations:** Secretary


### NEW YORK COUNTY VETERINARY MEDICAL ASSOCIATION.

THE regular meeting was held on Tuesday evening, December 3, 1895, at 8.30 o'clock, with the President, Dr. Huidekoper, in the chair. On roll-call, the following members responded to their names, viz.: Drs. Amling, Bieser, Bretherton, C. C. Cattanach, J. J. Cattanach, J. S. Cattanach, J. S. Cattanach, Jr., Dickson, Delaney, Dair, Ellis, Ferster, Farley, Giffen, Gill, Glover, Huidekoper, Hanson, Jackson, Johnson, Knott, Loomes, Lellman, Machan, MacKellar, Neher, O'Shea, Parsons, Ryder, Richards, Sielman, Turner (32). The minutes of the last meeting were read and approved.

Dr. Gill, chairman of the Board of Censors, reported that the charges against Dr. S. K. Johnson, not being in writing, were not made in the regular order according to the by-laws, and were referred back to the Association. The charges against Dr. R. W. Finlay were laid over until the next meeting for further evidence. Moved and seconded that the report be accepted. Carried.

Dr. F. W. Turner read a short paper on the necessity of having ambulance-service, etc., for Harlem, and suggested several very material improvements and changes that ought to be recommended to the American Society for the Prevention of Cruelty to Animals. A general discussion followed the paper, in which a majority of the members took part.

Judiciary Committee, Dr. O'Shea, Chairman, reported progress.

Committee on Certificates: Dr. Turner, Chairman, stated that the certificates, a copy of which was exhibited, were ready and would be signed and delivered at the next regular meeting. Moved and seconded, that each member's dues and assessments be paid before he could receive the certificate of the Association; carried.

No new members; no applications for membership.

This being the annual meeting, the election of officers for the ensuing year was next in order. Moved and seconded, that a recess of fifteen minutes be taken; carried. Nominations for President being next in order, the following nominations were made, viz., Drs. Huidekoper, Hanson, and Giffen. Moved and seconded, that the nominations close; carried. For Vice-President, Dr. Thomas Giffen was nominated. Moved and seconded, that the nominations close; carried. For Secretary, the following nominations were made, viz., Drs. Ellis, Ryder, C. C. Cattanach, and Hanson. Moved and seconded, that the nominations close; carried. For Treasurer, the following were nominated, viz., Drs. Neher, Parsons, and C. C. Cattanach. Moved and seconded, that the nominations close; carried. The election now took place with the following result: President, Dr. R. S. Huidekoper; Vice-President, Dr. Thomas Giffen; Secretary, Dr. R. W. Ellis; Treasurer, Dr. C. C. Cattanach. The President then addressed the members and thanked them for the honor bestowed upon him, after which the new officers were introduced to the members, each in turn making a short address.

Moved and seconded, that an Auditing Committee be appointed to examine the Treasurer's report; carried. The President appointed, as such committee, Drs. Hanson and J. S. Cattanach. After examining the report, they reported the same correct. The report showed a balance in the treasury of $13.18.

Moved and seconded, that a vote of thanks be extended to Dr. Ryder, the retiring Secretary; carried. Moved and seconded, that the meeting adjourn; carried.

J. E. Ryder, D.V.S.,
Secretary.

